# Fools vs. Yellow Fever Inoculation

**Published:** 1886-09

**Authors:** 


### FOOLS VS. YELLOW FEVER INOCULATION.

It has been asserted by a prominent scientist that "every man who has tried no experiment in his life is a fool," and as a consequence that no fool ever tried an experiment.

We are not responsible for the appositeness of this designation to certain medical men and members of Congress who have been prominent in opposing the investigation of the claims of inoculation as a prophylactic measure against yellow fever.

Facts demonstrating its efficacy have been ignored by theorists, who will not yield their peculiar views on the subject to adopt the results of actual experiment. Having impressed their false interpretation of bacteriology upon others, they are incapable of recognizing the inductive process of reasoning from known data to great general principles in science. They claim to have been instrumental in staying the proceedings of Congress towards a thorough examination of the process practiced successfully by Domingos Friere in Rio de Janeiro upon a scale which challenges investigation by the Societé de Biologie of Paris.

If the report of M. Rebourgeon, after a practical observation of two years in Rio de Janeiro, endorsing and confirming the experiments of Friere, was entitled to the consideration of such a commission as Brown-Sequard, Conil and others of like distinction in France, we may well understand that the rush-light of would-be guides of the blind in this country must pale ere long before the elucidations of this subject.

When the residents of our exposed Southern seaports shall come to realize that this opposition to appointing a commission for the investigation of the claims of yellow fever inoculation has been fostered by little, petty, personal animosities, and they become satisfied of the trustworthiness of this prophylactic measure, these upstart fledglings of bacteriology must hide their heads in the sand as the ostrich does when pursued by the hunter.

